# The Negro with a Tail

**Published:** 1883-12-20

**Authors:** 


### *THE NEGRO WITH A TAIL.*

The case of Prof. Johnson, relative to a singular tumor which he recently removed from a negro, at the clinic of the Southern Medical College in this city, is a feature of interest in the present number of our Journal. The negro had attracted much attention, and the Darwinites in our community were jubilant over this case as furnishing indubitable proof of the descent of the negro from the monkey tribe. It is unfortunate, however, that this negro's tail does not exactly come out from the right place.

